# The Book World of Medicine and Science

**Published:** 1902-06-14

**Authors:** 


### The Book World of Medicine and Science.

Clinical Methods: A Guide to the Practical Study of Medicine. By Robert Hutchison, M.D., M.R.C.P., and Harry Rainy, M.A., F.R.C.P.Ed., F.R.S.E. With upwards of 150 illustrations and eight coloured plates. New edition. (London: Cassell and Co. 1902. Price 10s. 6d.)

Although it is only a short time since the publication of the first edition of this book, which has already gone through several reprints, a new edition has been called for in which many alterations have been made, and to which much new knowledge has been added. Chapter V., which deals with the clinical examination of the blood, has been well brought up to date, and not only contains details as to obtaining and staining blood films, estimating hæmoglobin, and counting the various cellular elements, but gives full directions in regard to the examination of the blood for filariæ and malaria parasites. The series of changes occurring in the latter, of which good coloured illustrations are given, are well described. In regard to the specific gravity of the blood, the authors say that this is "always in proportion to the amount of hæmoglobin—a low specific gravity means little hæmoglobin. The ratio is so constant that one can tell the percentage of hæmoglobin by taking the specific gravity." This is a statement of considerable interest. The chapter on the nervous system has been well brought up to date; but, as the authors say, much recent knowledge of the mechanism of the central nervous system is still in an unsettled condition, and we shall not be surprised if, when the next edition is brought out, still further alterations may have to be made. As it stands, however, it fairly represents modern conceptions. It is a complicated subject well worked out. Clinical bacteriology is the subject of another chapter in which new knowledge has led to a good deal of alteration and improvement. So far as can be done in the small number of pages at their disposal the authors have done well, but we fear that unless a student has gone through a practical course much of the bacteriological lore here condensed will be wasted upon him. Throughout the rest of the book many minor alterations have been made, and much new knowledge has been interpolated, and we have no hesitation in recommending this new edition as a capital presentment of the clinical methods now in use at our best hospitals.

Selected Essays and Addresses. By Sir James Paget. Edited by Stephen Paget, F.R.C.S. (London: Longmans, Green and Co. 1902. Price 12s. 6d. net.)

In a manner it may be said that this book is issued *in memoriam*; and a good memorial it makes, recalling to those of us who knew him of the measured sentences and well considered judgments of one who for a long series of years was the most trusted of advisers. These lectures, essays, and addresses by Sir James Paget have, as the preface tells us, been republished not for "general reading," but for the special use of the medical profession, the lectures chosen being those which were most closely connected with his clinical studies and with his later work in surgical pathology. Among them we notice that old lecture delivered in 1867, in which, rather to the astonishment of his more narrow-minded brethren, he pointed out that "bone-setters" sometimes succeeded in curing even after careful professional treatment had failed, and in which he showed the *rationale* of these successes and the proper treatment which ought to be applied in cases of like nature. We are all accustomed now to see massage used in the treatment of recent sprains, but in 1867 few surgeons were prepared to recommend such a measure, although similar proceedings had many times been introduced only to be abandoned. Even Sir James Paget in bringing it before the profession, qualified his advice by saying "I suspect that it sometimes does no good, and sometimes does harm enough to disgust a prudent surgeon." It was, however, as he pointed out, among old sprains that the bone setters, especially those who combined rubbing and shampooing with their "setting," gained their chief repute, "and not without some right." "Among 'old sprains' are not a few cases in which a joint, after long treatment, remains or becomes habitually cold. It is generally stiffish and weak, sensitive, aching after movement, or in the evening or at night, sometimes swollen, puffy, or œdematous, but not with an 'œdema calidum.' Whatever else it is, it is cold, or at the most not warmer than the healthy fellow joint. Among these cold joints bone setters and rubbers gain great repute; and all the more because they often get the cases after the patients have become tired and discontented with a rather over-careful surgery." Then we have his celebrated address on "Stammering with other Organs than those of Speech," and among others which have become landmarks in surgical history are his admirable lectures on "Nervous Mimicry," on "Disease of the Mammary Areola preceding Cancer of the Mammary Gland," and on "Osteitis Deformans." If one would see how wide was the grasp which Sir James Paget had of the subjects which he studied, one must read the remarkably interesting address on "Elemental Pathology," delivered at Cambridge in 1880, in which he traced the consequences of injury and disease in the structures of plants. Addresses on "Theology and Science," on "Temperance and Alcohol," and other subjects, followed by a series of most interesting "Studies of Old Case Books," make up a volume which will not only be treasured as a memento of a great man and a most interesting personality, but may be read with interest and advantage by many of the younger generation in whose minds the name "Paget" is chiefly associated with the striking work which he did in the realms of pathology and surgery.

The Pocket Gray or Anatomists' Vade Mecum. Fifth Edition. Revised and edited by C. H. Fagge, M.D. (London: Baillière, Tindall and Cox. 1901. Pages 250. Price 3s. 6d. net.)

Dr. Fagge has, in this edition of the late Mr. Cotterell's well-known little work, corrected some mistakes and amplified some descriptions. At the same time he has endeavoured to preserve its original features and its character as a pocket volume. The "Pocket Gray" is too well known to need description. Its popularity seems well maintained, and we have no doubt that for years yet to come it will be welcomed by students who care to tackle anatomical facts in 'tabloid" form, and—to continue the metaphor—without the adventitious but often useful addition of morphological correctives, embryological adjuvants, and literary vehicles.

Desmonde, M.D. By Henry Willard French. (London: T. Fisher Unwin. 1901. Price 2s. 6d.)

It is difficult to understand why a book like this should have been written. By aid of hypnotism (interpreted in such a fashion as to be capable of doing anything the author happens to find convenient) and opiates, drink and rage, Mr. Willard French has managed to fill out a couple of hundred pages with a fantastic string of strange imaginings. We suppose that the excuse will be that it is a study in psychology. A study of the people who enjoy the reading of such stuff might be more interesting.

